# Improved affinity at the cost of decreased specificity: a recurring theme in PDZ-peptide interactions

**DOI:** 10.1038/srep34269

**Published:** 2016-10-03

**Authors:** O. Andreas Karlsson, Gustav N. Sundell, Eva Andersson, Ylva Ivarsson, Per Jemth

**Affiliations:** 1 Department of Medical Biochemistry and Microbiology, Uppsala University, BMC Box 582, SE-75123 Uppsala, Sweden; 2 Department of Chemistry-BMC, Uppsala University, Box 576, SE-751 23 Uppsala, Sweden

## Abstract

The E6 protein from human papillomavirus (HPV) plays an important role during productive infection and is a potential drug target. We have previously designed a high affinity bivalent protein binder for the E6 protein, a fusion between a helix from the E6 associated protein and PDZØ9, an engineered variant (L391F/K392M) of the second PDZ domain from synapse associated protein 97 (SAP97 PDZ2). How the substitutions improve the affinity of SAP97 PDZ2 for HPV E6 is not clear and it is not known to what extent they affect the specificity for cellular targets. Here, we explore the specificity of wild type SAP97 PDZ2 and PDZØ9 through proteomic peptide phage display. In addition, we employ a double mutant cycle of SAP97 PDZ2 in which the binding kinetics for nine identified potential cellular peptide ligands are measured and compared with those for the C-terminal E6 peptide. The results demonstrate that PDZØ9 has an increased affinity for all peptides, but at the cost of specificity. Furthermore, there is a peptide dependent coupling free energy between the side chains at positions 391 and 392. This corroborates our previous allosteric model for PDZ domains, involving sampling of intramolecular energetic pathways.

Persistent infection by high-risk human papillomavirus (HPV) can lead to cancer^1^. This is an indirect consequence of the combined actions of the HPV E6 and E7 proteins that are expressed during productive infection^2^. The HPV E7 protein binds to the retinoblastoma protein RB^3^, and target the protein for proteasomal degradation^4^. This would normally invoke a p53 dependent response, but it is blocked by the HPV E6 protein that targets p53 for degradation^5^,^6^. This gradually generates a genomic instability that can progress to cancer^7^. The continued expression of both HPV E6 and E7 is of equal importance for the proliferation of transformed cells^8^, designating the HPV E6 as a promising drug target^9^. The HPV E6 protein of high-risk papillomavirus types, such as HPV18 E6, has a C-terminal sequence that binds to several PDZ (*p*ostsynaptic density protein-95/*d*iscs large/*z*onula occludens-1) domains^10^,^11^. The hijacking of PDZ containing proteins by high-risk HPV E6 protein is linked to deregulated cell polarity and cell proliferation^12^. One of the PDZ proteins affected by HPV18 E6 is the Synapse Associated Protein 97 (SAP97, also called Dlg1)^10^,^11^, and the interaction targets SAP97 for proteasomal degradation^13^. SAP97 has three PDZ domains, of which all are known for binding to the HPV18 E6 peptide^14^,^15^

PDZ domains consist of roughly 90 amino acids and typically recognize short C-terminal motifs in proteins^16^,^17^. The carboxylate group is bound by a conserved GLGF loop while the rest of the motif aligns as a β-strand to form an extended β-sheet with the PDZ fold (see Fig. 1). PDZ domains are divided into classes based on their ligand binding specificity, originally defined by the four amino acid residues at the C-terminus of the protein ligand, at positions p-3, p-2, p-1 and p0, respectively. The main classes are class 1 (x-(T/S)-x-Φ) and class 2 (x-Φ-x-Φ), where x indicates any amino acid and Φ is a hydrophobic residue^18^,^19^. More fine-grained classification systems have been derived by taking into account interactions throughout the binding pocket. Indeed the PDZ domains have been grouped into at least 16 different classes^20^,^21^. In this study, we focus on the second PDZ domain of SAP97 that has a preference for RETxV containing peptides^15^,^21^, which matches the C-terminal motif of the HPV18 E6 C-terminus (RRRETQV).

Inhibitors of high-risk HPV E6 proteins may be used for diagnostics and treatment of HPV-induced cancers^9^. We previously designed a high affinity binder for the E6 protein of high-risk HPVs. This ‘PDZbody’ targets the E6 proteins by using two distinct binding interfaces, a “LxxLL” motif and a SAP97 PDZ2 variant (PDZØ9) optimized for high-affinity HPV18 E6 binding. PDZØ9 is an engineered variant of a pseudo wild type of the second PDZ domain of SAP97 (pWT PDZ2). pWT PDZ2 has two mutations as compared to wild type SAP97 PDZ2 (I342W and C378A) and is the variant used in our previous studies on the binding of SAP97 PDZ2^22^,^23^,^24^. PDZØ9, in turn, was selected from a library of pWT PDZ2 variants using phage display and it carries two additional mutations as compared to pWT PDZ2, L391F and K392M. The L391F mutation is located in the p0 hydrophobic binding pocket, and is expected to make direct contacts with bound peptide. This position is known to contribute to the specificity of PDZ domains for the p0 residue^25^, while the significance of the K392 mutation is less obvious.

In the present study we investigate how the two mutations modulate the affinity and specificity of PDZØ9 with preferred targets in the human proteome. We obtain a set of preferred peptides for SAP97 PDZ2 and PDZØ9 by using the respective protein as bait in selections against a human phage peptidome, a phage library that displays all C-terminal peptides of the human proteome. Identified ligands are used in dedicated binding experiments, which reveal that the high affinity of PDZØ9 is accompanied by a loss of specificity. Furthermore, a double mutant cycle analysis on PDZØ9 reveals a peptide-dependent energetic coupling between the two mutated residues, situated in the second alpha helix of PDZØ9. These results are in line with previous experiments suggesting dynamic, rather than conserved energetic networks in the PDZ domain family^26^,^27^.

## Results

First, we used proteomic peptide-phage display (ProP-PD)^15^ to identify preferred binding peptides in the human proteome for SAP97 PDZ2 and PDZØ9. Among identified ligands, we selected nine representative peptides. We then used the precision of stopped-flow spectroscopy to address (*i*) the specificity for the selected peptides benchmarked with a peptide corresponding to the C-terminus of HPV18 E6, and (*ii*) the presence of energetic coupling (‘communication’) between the residues at positions 391 and 392 in PDZØ9.

### Choosing nine peptides that represent potential ligands within the SAP97 PDZ2 interactome

In order to choose a set of peptides that represents the sequence space for C-termini of potential ligands within the SAP97 PDZ2 interactome, we performed ProP-PD selections using pWT PDZ2 and PDZØ9, respectively, as bait in separate selections. pWT PDZ2 and PDZØ9 were first confirmed to be well folded under the conditions in this study through circular dichroism spectroscopy (see Materials and Methods and Supplementary Fig. S1). The peptide-phage library used in the selections consisted of 50,549 heptapeptides representing the C-terminal sequences of the human proteome, fused to the C-terminus of the major coat protein pVIII of the M13 phage. This library was developed and successfully used for the rapid isolation of potential cellular PDZ domain targets, as previously reported^15^.

A plateau in terms of enrichment of binding phages was reached after the third round of selection as judged by pooled phage enzyme-linked immunosorbent assay (ELISA; data not shown). Individual binding clones were isolated and sequenced (see Table 1). For PDZØ9, we obtained 17 unique peptide sequences from 76 sequenced clones that matched C-termini from human proteins. For pWT PDZ2, 9 unique sequences were retrieved from 71 sequenced clones. Seven of the selected peptides were found in selections against both pWT PDZ2 and PDZØ9, with the KRKETLV peptide displaying the highest prevalence among the sequenced phages in both datasets. Strikingly, all peptides preferred by pWT PDZ2 have a Val at position p0, while the PDZØ9 ligands contain Val/Leu/Ile at this position. Thus, PDZØ9 has a more relaxed specificity for the residue at p0 as compared to pWT PDZ2.

Several of the identified ligands have previously been reported to interact with either SAP97 or with SAP97 PDZ2 specifically. Indeed, 12 of the 19 selected ligands have been proposed as SAP97 PDZ2 ligands through ProP-PD. Five of the ligands selected by both SAP97 PDZ2 and PDZØ9, and two ligands selected as ligands only for PDZØ9, have been confirmed as SAP97 binding partners through methods such as immunprecipitation. In addition, the VSKETPL peptide, representing the p38γ protein (see Supplementary Table S1), has to our knowledge not been reported previously to interact with SAP97 PDZ2 but to SAP97 PDZ1 and PDZ3^28^. It is therefore clear that the ProP-PD selections enriched peptides of C-termini originating from potentially biologically relevant targets within the SAP97 PDZ2 interactome. By analysing the data available in the human protein atlas^29^ we find that the proteins that host the identified peptides all overlap with SAP97 in terms of tissue expression and subcellular localisation (see Supplementary Table S1). It is thus plausible that SAP97 interacts with the identified proteins in a cellular context.

Based on the composition of amino acids and prevalence among the survivors in both of the selections, nine peptides were chosen to represent the sequence space for such C-termini of potential ligands (see Table 1).

### The L391F mutation alone provides the increased affinity that PDZØ9 displays for all selected peptides

The set of nine selected peptides (see Table 1) and the HPV18 E6 peptide RRRETQV, which PDZØ9 was engineered to have high affinity for^22^, were subjected to binding studies with pWT PDZ2, pWT PDZ2 L391F, pWT PDZ2 K392M, and PDZØ9, respectively, using stopped-flow spectroscopy. The basic experimental parameters determined were the rate constants of association (*k*_on_) and dissociation (*k*_off_) for each peptide and for all PDZ variants, respectively, in a procedure described in Fig. 2 for the interaction between PDZØ9 and the EKKHTLL peptide as an example.

Our reported K_d_ values (=*k*_off_/*k*_on_) are to our knowledge the first quantification of the strength of interaction between SAP97 PDZ2 and any of the nine selected peptides (see Fig. 3 and Supplementary Table S2). Analysis of the K_d_ values demonstrates that pWT PDZ2 binds with affinities of a few μM to all of the nine selected peptides, which is in agreement with those of 1–50 μM typically displayed by PDZ domains and their natural ligands^30^.

The PDZØ9 affinity of 0.40 μM for the C-terminus of HPV18 E6 is the highest measured in the study, and can only be matched by the NET1 (ARGH8_HUMAN) peptide KRKETLV. This suggests that HPV18 E6 has evolved to have a high affinity for this PDZ domain in comparison to proteins within the SAP97 PDZ2 interactome. Furthermore, PDZØ9 displays an increased affinity for all 10 peptides investigated. Interestingly, this increase in affinity is afforded by the L391F mutation alone. The K392M mutation has either no effect, or, as for EKKHTLL, VSKETPL and TSRETDL, results in a slight decrease in affinity (see Fig. 3).

### The magnitude of energetic coupling between the two mutated residues in PDZØ9 depends on the peptide that the protein interacts with

Next we used the kinetic dataset to investigate in more detail the interplay between the two positions 391 and 392. In order to probe the intramolecular interaction between them in PDZØ9 we adopted a methodology known as a double mutant cycle, originally developed by Fersht and co-workers^31^,^32^. A double mutant cycle makes it possible both to determine if there is an interaction between any two amino acid residues or not, and to quantify the interaction as the coupling free energy (ΔΔΔG_c_) and a detailed description of this has previously been reported^26^. Within this context the term coupling is used instead of interaction, since the interaction may be indirect through other residues in the protein rather than a direct binding between the side chains. The ΔΔΔG_c_ is conventionally calculated for the overall binding reaction at equilibrium using the four K_d_ values determined with each peptide (see Materials and Methods, Equation 2). In the present study we also calculated the coupling free energy for the transition state, ΔΔΔG_c_^TS^ using the *k*_on_ values (see Materials and Methods, Equation 3).

Among the ten analysed peptides, seven (including the HPV18 E6 peptide) displayed coupling free energies ΔΔΔG_c_ between F391 and M392 close to zero (see Fig. 4 and Supplementary Table S3). On the other hand, for the peptides with a Leu at the C-terminus, EKKHTLL, VSKETPL and TSRETDL, there is a substantial coupling between F391 and M392 in PDZØ9. Roughly half of this coupling free energy originates at the transition state for binding. These are the same three peptides for which the K392M single mutation in pWT PDZ2 resulted in a slight decrease in affinity. Since the L391F mutation alone instead yielded K_d_ values comparable to that for PDZØ9, the measurable coupling enables a scenario where the presence of L391F compensates for the effect of K392M. In conclusion, the degree of coupling between F391 and M392 in PDZØ9 at the transition and bound states clearly depends on the peptide that the protein interacts with.

### PDZØ9 displays a reduced specificity for the C-terminus of HPV18 E6

PDZØ9 was originally selected for the C-terminus of HPV18 E6, which corresponds to the peptide RRRETQV, with the aim of improving both affinity and specificity. However, the phage display data indicate that the specificity of PDZØ9 has been compromised. Indeed, by constructing sequence logos that visualize the specificity for each peptide ligand position for pWT PDZ2 and PDZØ9, respectively, it is clear that the main difference between the preferences of the two proteins is that Val is the only residue likely to be found at the C-terminus among the peptides selected for pWT PDZ2, while Val and Leu are equally likely to be found at the corresponding position among the peptides selected for PDZØ9 (see Fig. 5a).

To estimate the loss of specificity of PDZØ9 we further analysed the K_d_ values in our data set with regard to the specificity for the RRRETQV HPV18 E6 peptide in comparison with the nine ProP-PD peptides using the following expression.


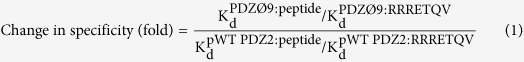


In this equation, the change in specificity for PDZØ9 relative to pWT PDZ2 is calculated for each of the peptide ligands in the study, and with the RRRETQV peptide as the benchmark. According to this expression, a value of 1 means that there is no difference in specificity between PDZØ9 and pWT PDZ2 for peptide X relative to the HPV18 E6 peptide RRRETQV. A value >1 means that PDZØ9 has gained specificity relative pWT PDZ2 for the RRRETQV peptide in comparison with the ProP-PD peptide.

**Figure 1:**
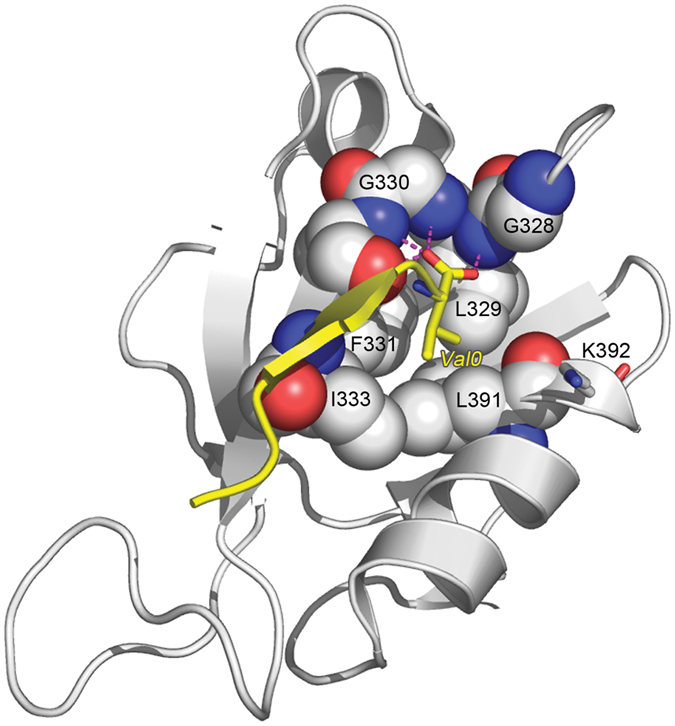
Model of SAP97 PDZ2 with bound ligand. X-ray crystal structure of SAP97 PDZ2 in complex with a heptapeptide (RRRETQV) corresponding to the C-terminus of HPV18 E6 (Protein data bank code: 2I0L). The peptide binds as an anti-parallel β-strand in a so-called β-augmentation process with its carboxyl group situated in proximity of the main chain nitrogens in the highly conserved GLGF-loop (highlighted residues G328-F331), thus participating in a network of hydrogen bonds (dashed lines in magenta)^41^. This is defined as the canonical binding mode for PDZ domains. The side chains of L329, F331, I333 and L391 form a hydrophobic pocked that interacts with the side chain of *Val0* in the bound peptide. *Val0* in the peptide is numbered according to the convention in the PDZ field and corresponds to residue 158 in the HPV18 E6 protein. This position is important for the affinity and specificity of the complex. The SAP97 PDZ2 residues L391 and K392 are substituted for Phe and Met, respectively, in PDZØ9. We here investigate how these two residues influence the specificity for the HPV18 E6 protein in comparison with potential cellular binding partners within the SAP97 PDZ2 interactome and the intramolecular interaction between them upon ligand binding. The figure was generated using PyMOL (http://www.pymol.org).

**Figure 2:**
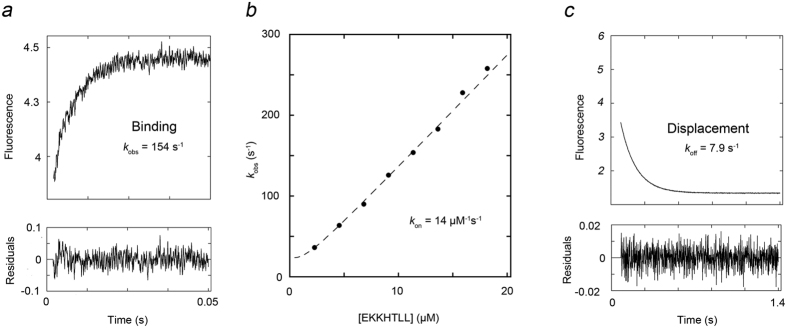
Determination of the two rate constants *k*_on_ and *k*_off_ for PDZØ9 binding to the EKKHTLL peptide. *k*_on_ and *k*_off_ were determined in separate experiments using stopped-flow spectroscopy and monitoring the change in Trp fluorescence upon binding. (**a**) The experimental trace was obtained by averaging of three individual experiments in which 11 μM of the EKKHTLL peptide was mixed with 1 μM PDZØ9. The trace was fitted to a single exponential function to obtain the observed rate constant, *k*_obs_. (**b**) Observed rate constants were plotted *versus* different concentrations of EKKHTLL to obtain the association rate constant *k*_on_ as the slope of the curve (see Supplementary Figure S2 for the corresponding curves for all PDZ variants and peptides included in this study). (**c**) In a displacement reaction a pre-formed complex between PDZØ9 (1 μM) and the EKKHTLL peptide (2 μM) was mixed with a large excess of dansyl-labeled peptide corresponding to the C-terminal six residues of HPV18 E6 (150 μM). The dansyl-labeled peptide competes for binding to PDZØ9, and its binding results in a large change in dansyl fluorescence. At high concentrations of dansylated peptide, none of the dissociated EKKHTLL will re-bind, and fitting to a single exponential function returns a *k*_obs_ value which is equal to the dissociation rate constant, *k*_off_, of the pre-formed complex. The initial part of the trace corresponds to binding of free PDZØ9 to the dansyl-labeled peptide (*k*_obs_∼1000 s^−1^) and is not included in the fit. See Fig. 3 and Supplementary Table S2 for rate constants and K_d_ values for all PDZ variants and peptides included in this study.

**Figure 3:**
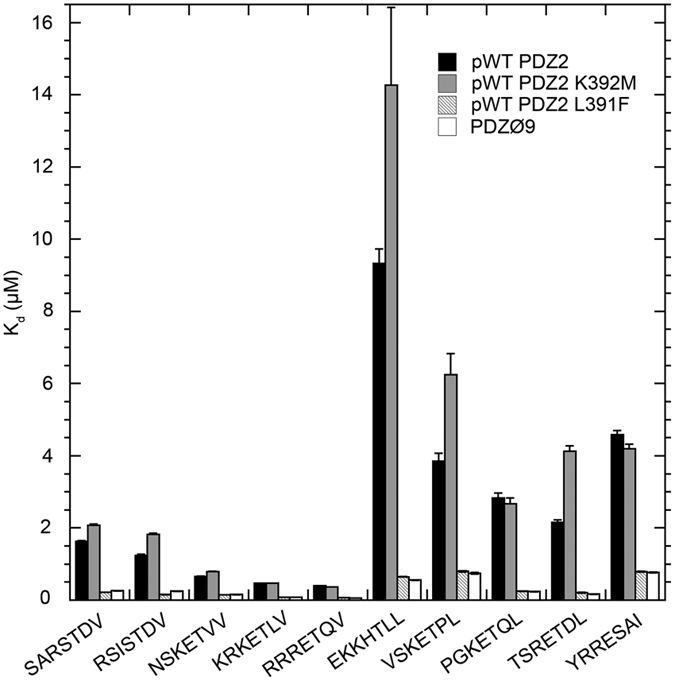
Histogram of equilibrium dissociation constants for the binding between PDZ variants and peptides, which represent potential ligands within the SAP97 PDZ2 interactome. The K_d_ value for the PDZ/peptide interaction was determined by the ratio of *k*_off_/*k*_on_. Each column represents a unique K_d_ value for a PDZ/peptide interaction as defined in the figure. The displayed error bars are the propagated standard error from *k*_on_ and *k*_off_. All K_d_ values can be found in Supplementary Table S2.

**Figure 4:**
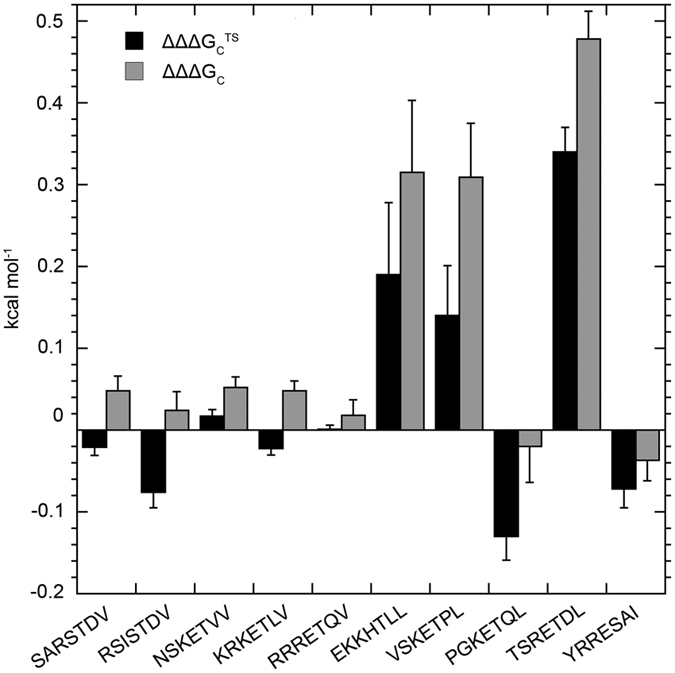
Histogram of coupling free energies between F391 and M392 at the transition state and bound state of PDZØ9 binding to peptides representing potential ligands within the SAP97 PDZ2 interactome. The intramolecular coupling upon binding between the side chains F391 and M392 in PDZØ9 was addressed quantitatively with a double mutant cycle analysis in which the coupling free energy of binding was calculated for each of the nine peptides at both the transition state, ΔΔΔG_c_^TS^, and at the bound state, ΔΔΔG_c_. The error bars are the propagated standard error from the K_d_ values. All ΔΔΔG_c_ values can be found in Supplementary Table S3. See the Materials and Methods section for a description of the calculations.

**Figure 5:**
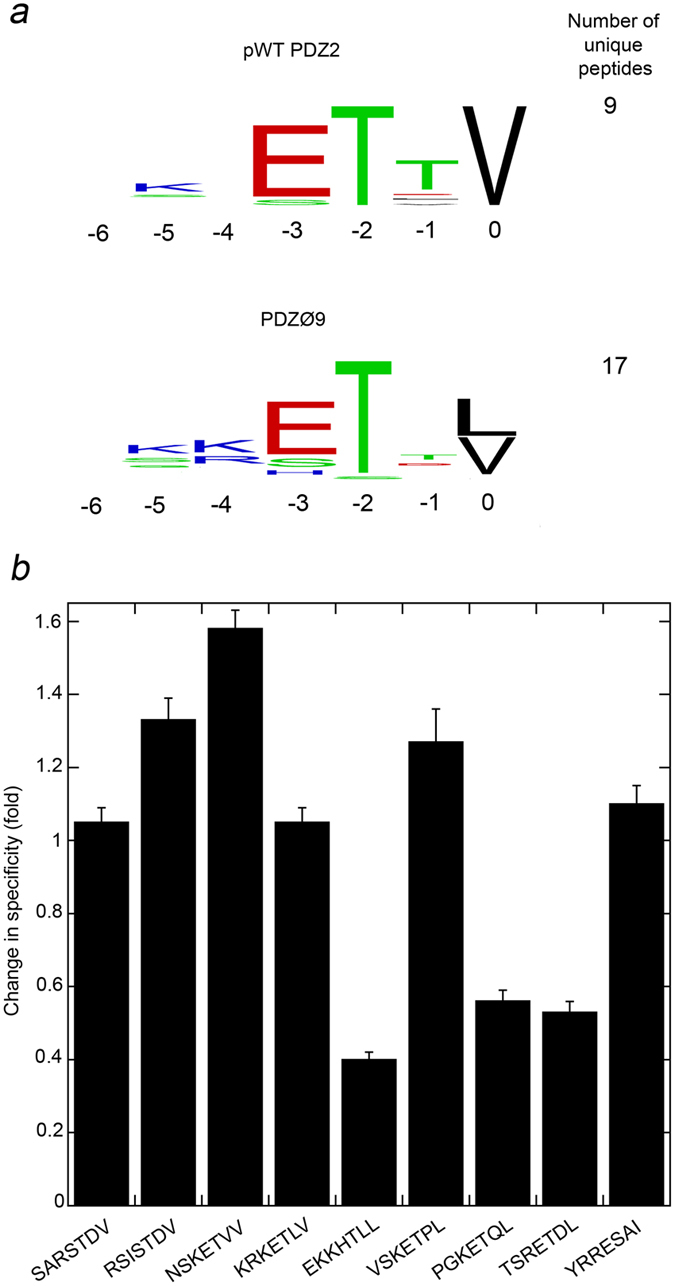
Change in specificity for PDZØ9 relative to pWT PDZ2. (**a**) Sequence logos that visualize the specificity for each ligand position for pWT PDZ2 and PDZØ9, respectively (generated by using WebLogo^42^). They are constructed from the respective alignment of the nine and seventeen unique peptide sequences obtained from ProP-PD selections. A large single letter means that the sequence alignment contains no other residue at that position, and the protein is therefore more specific for that particular residue at that ligand position. At position 0, PDZØ9 tolerates Val and Leu to an equal extent, while pWT PDZ2 is more specific for Val. PDZØ9 therefore displays overall reduced ligand specificity. (**b**) Each column in the histogram is the change in specificity for PDZØ9 relative to pWT PDZ2 calculated for each particular C-terminal peptide with the RRRETQV peptide as the benchmark. A value of 1 means that there is no change in specificity. The error bars are the propagated standard error from the K_d_ values. For numbers on the calculated specificity values see Supplementary Table S2.

The calculations demonstrate that for six of the peptides, the specificity is either only slightly increased (in one case 1.6-fold, NSKETVV) or virtually unchanged. For the remaining three peptides, EKKHTLL, TSRETDL and PGKETQL, PDZØ9 displays a clear loss in specificity for the RRRETQV peptide (see Fig. 5b and Supplementary Table S2). These three peptides have in common a Leu at their C-terminus. The structural reason behind this observation could originate in the strength of the interaction between the backbone of the peptide residues and the main chain of the β-strand that it interacts with in a β-augmentation fashion (see Fig. 1). It has previously been suggested that SAP97 PDZ2’s discrimination between a Val and a Leu at p0 in the peptide is due to the hydrophobic pocket, which better accommodates a Leu, resulting in an outward translocation of the peptide backbone that weakens its interaction with the main chain of the adjacent β-strand in the PDZ domain^33^. Since the increased affinity of PDZØ9 for all peptides measured is due to the L391F mutation in the hydrophobic pocket, a Phe at position 391 likely contributes to the favourable free energy of binding first of all through increased hydrophobic interaction with the p0 side chain and/or increased burial of hydrophobic surface. Secondly, it could leave more degrees of freedom for a Leu side chain at p0, allowing for optimization of the interactions outside the hydrophobic pocket during the β-augmentation process and a relatively higher increase in affinity. The affinity could in such way be increased at the cost of specificity for the RRRETQV peptide. However, the Pro side chain at p-1 in the VSKETPL peptide could impose structural constraints on the peptide backbone that eliminates the cost in specificity according to the scenario described above. Finally, the fact that three out of four peptides with a Leu at p0 display a substantial loss in specificity for the RRRETQV peptide, and PDZØ9’s generally increased affinity for all selected peptides, explain why ProP-PD with PDZØ9 as bait selected nearly twice as many unique peptides than using pWT PDZ2 as bait.

## Discussion

Specific inhibitors of high-risk HPV E6 proteins have the potential to be used as experimental tools and even drugs against HPV-induced cancers^9^. As a part of our design of a high-affinity HPV18 E6 binder, we engineered PDZØ9 through *in vitro* evolution to have a six fold increase in affinity for the HPV18 E6 peptide in comparison to the wild-type SAP97 PDZ2^22^. Here we investigated the specificity of PDZØ9 towards a panel of peptides and also the effect of the two mutations in PDZØ9, L391F and K392M in the binding reaction.

As PDZØ9 was optimized for binding to the RRRETQV peptide it was assumed that the increased affinity came hand-in-hand with increased specificity for the given peptide. However, here we find through peptide phage display that PDZØ9 has reduced capability to discriminate between a Val and a Leu at position 0, despite being engineered for high affinity for the HPV18 E6 peptide, which has a Val at this position. The kinetic binding analysis demonstrates that the protein has gained affinity for the HPV18 E6 peptide, as well as all other peptides tested. Furthermore, the results demonstrate that PDZØ9 displays an increased affinity for all peptides as compared to pWT PDZ2 due to the L391F mutation in the peptide binding site. Such a gain in affinity at the cost of specificity can be linked to the “negative selection” model, according to which binding site residues may be optimized for affinity and/or for maximized specificity against other ligands^34^,^35^. In this sense L391 in the context of wild type SAP97 PDZ2 may be suboptimal for binding, but serves as a gatekeeper residue against undesired peptides.

A similar result supporting the contradicting demands of affinity versus specificity in PDZ domains was previously found by Ernst *et al.* using a phage library displaying Erbin PDZ variants that was put under selective pressure for affinity against a set of peptides^36^. Our experiments thus confirm that an increase in both affinity and specificity in PDZ peptide interactions is difficult to achieve with mutations only in the PDZ peptide-binding pocket. However, a recent study on Erbin PDZ suggests that specificity could be achieved with (i) inclusion of positions distal from the binding pocket, combined with (ii) competitive binding during the selection process^37^. However, the study did not assess how the affinities were affected, and it therefore remains to demonstrate if an increase in both affinity and specificity for a certain PDZ domain target is possible to achieve with affinity based phage display experiments. Our HPV E6-binding PDZbody is based on an inclusion of a second binding interface (an E6AP helix), which should specifically increase the affinity for HPV E6^22^.

Finally, the detailed analysis of the effect of the two mutations in PDZØ9, L391F and K392M on the binding reaction showed that there is a significant energetic coupling between F391 and M392 in PDZØ9 and that this coupling is peptide dependent. This finding corroborates our previous allosteric model for PDZ domains, involving sampling of intramolecular energetic pathways for tuning binding selectivity^26^,^27^. The model suggests that different ligand positions (p0, p-1, etc.) are connected to different energetic pathways within the PDZ domain. These pathways originate at residues in direct contact with a certain ligand position and extend through the domain via a network of coupled residues. Peptides with a Leu at position p0 appear to ‘activate’ such a cryptic energetic pathway involving F391 and M392 in PDZØ9 whereas peptides with a Val do not. Thus, our results suggest that sampling of intramolecular energetic pathways in PDZØ9 is regulated by peptide identity.

## Materials and Methods

### Design of constructs

Pseudo wild type SAP97 PDZ2 (pWT PDZ2) is a variant used in several previous studies^22^,^23^,^24^,^27^ and constituting SAP97 residues 311–407 and the mutations I342W and C372A. pWT PDZ2 was also the ‘wild type variant’ from which the phage display library used to select PDZØ9 was designed. For the present study, pWT PDZ2 was subcloned into the same construct used for expression of PDZØ9, with an N-terminal His-tagged lipoyl protein domain which can be removed by thrombin cleavage^22^. In addition, constructs carrying either of the two mutations in PDZØ9 (L391F and K392M, respectively) were created with site directed mutagenesis, and denoted pWT PDZ2 L391F and pWT PDZ2 K392M, respectively.

### Protein Expression and Purification

All proteins were expressed and prepared for purification essentially as described previously^22^, with the exception that overexpression was induced with 0.2 mM isopropyl β-D-thiogalactopyranoside (IPTG). The first purification step was Ni-IMAC, which was performed using an ÄKTAxpress system (GE Healthcare) with a 1 ml HisTrap™ FF Crude column (GE Healthcare) followed by a HiPrep™ 26/10 buffer exchange column (GE Healthcare) equilibrated with 50 mM potassium phosphate pH 7.5, 400 mM NaCl. The method used was the system’s standard and the choice of buffers was according to the manufacturer’s recommendations. The collected sample, containing roughly 20 mg of protein, was digested with 10 units of thrombin at room temperature overnight and loaded on a Ni Sepharose™ 6 Fast flow (GE Healthcare) column followed in line by a 1 ml HiTrap™ Benzamidine FF column (GE Healthcare) to remove thrombin. The pure PDZ domain was collected in the flow through while the His-tagged lipoyl protein domain and residual impurities were retained on the Ni Sepharose column and eluted with imidazole. In preparation for Pro-PD the His-tagged lipoyl protein domain was purified from the eluate by changing the conditions to 50 mM Tris pH 8.5, 400 mM NaCl and reloading on a Ni Sepharose™ 6 Fast flow column on which the residual impurities were washed out using 50 mM Tris pH 8.5, 35 mM imidazole, 1 M NaCl before elution of His-tagged lipoyl protein domain with 50 mM Tris, 400 mM NaCl, 500 mM Imidazole pH 8.5. Undigested His-tagged lipoyl-PDZ fusion constructs of pWT PDZ2 and PDZØ9 were desalted into 50 mM potassium phosphate pH 7.5 after the Ni-IMAC. The collected sample was loaded onto a HR10 Source 30Q column (GE Healthcare) and eluted with a gradient of NaCl. To obtain pure lipoyl-PDZ the major peak from the HR10 Source 30Q chromatography was desalted and loaded on a 1 ml Resource Q column (GE Healthcare). Fractions containing pure lipoyl-PDZ were collected from the flow through of the Resource Q column step. All proteins were analysed with SDS-PAGE and mass spectrometry to verify purity and identity, respectively.

### Proteomic peptide-phage display selections

Peptide-phage selections against PDZØ9 and pWT PDZ2 were performed with a previously developed library of 50549 heptapeptides, representing all of the C-terminal sequences in the human proteome^15^. Peptides are fused to the C-terminus of the gene-8 major coat protein of M13 phage and hereby referred to as the naïve peptide library. Each of the two selections was performed twice. First, the two lipoyl-PDZ proteins were used to coat 96-well Maxisorp microtiter plates (NUNC) by incubation overnight at 4 °C (15 μg of protein in 100 μl of PBS per well). For the first round of selection, two wells were used for each PDZ, whereas a single well was used for the following selections. In parallel, wells were coated with lipoyl domain alone in order to remove nonspecific binders by a preselection step. The following day, wells were blocked with 200 μl of 0.5% BSA in PBS for 1 h at 4 °C, and washed four times with PT buffer [PBS, 0.05% Tween-20] before addition of phages. Phage pools representing the naïve peptide library were diluted 50-fold in PBS, precipitated with PEG/NaCl [4% PEG-8000 and 0.5 M NaCl] by incubation for 10 min on ice, pelleted by centrifugation (10 min, 16000 × g), and resuspended in PBS. For each selection round, 100 μl of resuspended phage was added to the preselection wells and incubated for 1 h at 4 °C before transfer to the target wells in which they were incubated for 2 h at 4 °C. The wells were washed five times with PT buffer and bound phages were eluted by direct infection of bacteria through the addition of 100 μl log-phase *Escherichia coli* Omnimax (Invitrogen) in 2TY, followed by 30 min incubation at 37 °C with shaking. Phage production was initiated by the addition of M13K07 helper phage (New England Biolabs) to a final concentration of 10^10^ phage/ml and continued incubation at 37 °C for 45 min. The cultures were then transferred to 10 ml 2TY containing 100 μg/ml carbenicillin, 30 μg/ml kanamycin, and 1 mM IPTG, and shaken overnight at 37 °C. The bacteria were pelleted by centrifugation (10 min, 5300 × g), and the phage-containing supernatant was transferred to a new tube in which phages were precipitated with PEG/NaCl as described above, pelleted by centrifugation (15 min, 5300 × g), and resuspended in 1 ml of PBS. The resuspended phages, hereby-denoted Ø_out_, were then used for the next round of selection. A total of five rounds were carried out and the enrichment of phages specific for the target was monitored by analysing aliquots of Ø_out_ in a phage ELISA performed as described below. For each of the five rounds of the respective selection, 10 μg of target protein and lipoyl domain were individually coated in parallel wells using the same type of plate, procedure, and blocking the following day, as described for the selection rounds above. When the 200 μl of blocking solution had been aspirated, 100 μl of resuspended phage was added and incubated for 30 min at 4 °C. The wells were then washed four times with PT buffer before 100 μl of anti-M13 antibody-HRP conjugate (GE Healthcare, 1:5000) in PBT buffer [PT buffer, 0.5% BSA] was added and incubated for 20 min at 4 °C. The unbound antibodies were washed out with four times of PT buffer and once with PBS, after which 100 μl of TMB substrate (KPL) was added. After 5 min the reaction was stopped by adding 100 μl of 0.6 M H_2_SO_4_ and the amount of product quantified by absorbance at 450 nm. The fold-value was calculated for each pair of wells with target protein and lipoyl domain. To further evaluate the presence of target-specific phages on the level of individual clones, after round three of selection, a colony phage Elisa was performed. 10 μl of Ø_out_ was allowed to infect 90 μl of log-phase *Escherichia coli* Omnimax, as described for elution of bound phages above, and diluted to obtain individual colonies when spread on LB plates containing 100 μg/ml of carbenicillin. The colonies were picked the following day and individual phage clones were propagated at 37 °C overnight using a 96-deep-well block (Axygen). Each well contained 350 μl of 2TY containing 100 μg/ml of carbenicillin and 10^10^ helper phages/ml. The bacteria were then pelleted by centrifugation (15 min, 5300 × g), and the supernatant was used in phage ELISA experiments. A fold-value >2 was considered as a target-specific phage clone. The DNA in the phagemid corresponding to the displayed heptapeptide was prepared for sequencing by first amplifying it from phage supernatant with PCR, using oligos that include annealing sites for M13 common primer (for sequencing reaction). The amount of product was quantified with electrophoresis. For a sample of PCR reaction mixture corresponding to a total of 150 ng of product, the remaining reaction substrates were digested with 0.2 units of Shrimp alkaline phosphatase (GE Healthcare) and 2 units of Exonuclease I (Affymetrix) for 30 min at 37 °C, followed by inactivation for 15 min at 80 °C, before submitted for sequencing.

### Stopped-flow Spectroscopy

All of the stopped-flow experiments conducted in order to measure the rate constants for the peptide/PDZ interactions, were performed on an SX-20 MV stopped-flow spectrometer (Applied Photophysics Leatherhead, UK) at 10 °C in 50 mM potassium phosphate buffer, pH 7.5. Fluorescence was monitored using the change in emission of Trp342 in the PDZ variants (excitation at 280 nm; emission at 330 ± 30 nm). The peptides were N-acetylated (Ontores Biotechnologies) and the concentration of the respective stock solution was determined with either absorbance, quantitative amino acid analysis or direct detect (EMD Millipore). The rate constants of association, *k*_on_, and dissociation, *k*_off_, were determined in separate experiments. For determination of *k*_on_ we mixed peptide (varied between 2–40 μM in different experiments) with PDZ (1 μM) and fitted the resulting trace of increase in tryptophan emission upon binding to a single exponential equation to obtain the observed rate constant *k*_obs_ at each peptide concentration. For each PDZ variant the *k*_obs_ values were plotted *versus* the concentration of peptide and fitted to the general equation for a reversible bimolecular interaction^38^,^39^ from which *k*_on_ was derived. The *k*_off_ was measured in a displacement experiment in which a pre-formed complex of PDZ variant (1 μM) and peptide (2 μM) was mixed with high concentrations of a dansylated RRRETQV-peptide (100, and 150 μM), which competes with the unlabelled peptide for binding to the PDZ. The observed decrease in tryptophan emission was fitted to either a single exponential equation to obtain *k*_obs_, or, when the *k*_off_ > 50 s^−1^ and the time frame of the measurement allowed detection of the fast binding (*k*_obs_ > 800 s^−1^) of dansylated peptide to free PDZ, to a double exponential equation in order to obtain a correct *k*_obs_ for the slow phase. The *k*_obs_ value at high peptide concentration is equal to the overall *k*_off_ value, as described previously^24^. For the displacements of complexes between pWT PDZ2 L391F or PDZØ9 with the PGKETQL peptide the time-resolved high resolution traces of change in fluorescence displayed a possible biphasic behavior, but the two *k*_obs_ values were not well separated (differed by a factor of 2.5). Therefore, fit to a single exponential function was used, which resulted in a *K*_d_ ( = *k*_off_/*k*_on_) that agreed with that determined by ITC under identical conditions, showing that the two-state assumption is valid also for these variants (see Supplementary Fig. S2).

### Calculation of coupling free energies

The coupling free energy of binding, ΔΔΔG_c_, was calculated by using the four K_d_ values determined for each peptide (Equation 2).


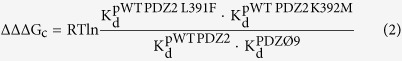


The coupling free energy at the transition state, ΔΔΔG_c_^TS^, was calculated using the *k*_on_ values determined with each peptide. (Equation 3).


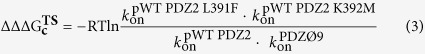


### Isothermal Titration Calorimetry (ITC)

ITC experiments were performed on an iTC200 (Malvern Instruments) at 10 °C in 50 mM potassium phosphate buffer, pH 7.5. The PGKETQL peptide was titrated into a solution of pWT PDZ2 L391F. The experiments were designed such that the C values were within 1–1000 (C value = N × [Protein]/K_d_, where N is the stoichiometry of the interaction, [Protein] is the molar concentration of protein in the cell and K_d_ is the equilibrium dissociation constant). The software provided by the manufacturer was used to determine the thermodynamic parameters of the peptide/PDZ interactions using nonlinear least square fitting assuming a 1:1 model. Since a clear saturation was reached in the experiments we corrected for the small heat of dilution by subtracting integrated peaks until a minimum in χ2 was obtained.

### Circular Dichroism (CD) Spectroscopy

Measurements of far-UV CD during thermal denaturation were recorded using a JASCO-810 CD spectropolarimeter, and a 0.1 cm cuvette. The temperature interval was 277–363 K and the signal recorded at 218 nm. The T_m_ was determined by fitting to the Gibbs-Helmholtz equation for a reversible two-state thermal denaturation^40^. Reversible denaturation was confirmed with identical far-UV spectra at 277 K before and after the denaturation. The protein concentration was 30 μM in 50 mM potassium phosphate pH 7.5.

## Additional Information

**How to cite this article**: Karlsson, O. A. *et al.* Improved affinity at the cost of decreased specificity: a recurring theme in PDZ-peptide interactions. *Sci. Rep.* **6**, 34269; doi: 10.1038/srep34269 (2016).

## Supplementary Material

Supplementary Information

## Figures and Tables

**Table 1:** Unique peptide sequences obtained from sequence analysis of binding clones obtained from ProP-PD selections using pWT PDZ2 or PDZØ9 as bait.

Peptide	Prevalence*		Uniprot	Reference***
	pWT PDZ2 (71)**	PDZØ9 (76)**		
KRKETLV	41	31	ARHG8_HUMAN	Pull-down^43^, ProP-PD^15^
RSISTDV	8	3	F163B_HUMAN	ProP-PD^15^
IKTETTV	7	1	RASF6_HUMAN	ProP-PD^15^
WKHETTV	5	4	GP125_HUMAN	IP^44^, ProP-PD^15^
NSKETVV	4		MARH3_HUMAN	ProP-PD^15^
KIKETTV	3	1	FRPD4_HUMAN	Pull-down^45^, ProP-PD^15^
WKSETTV	1	1	GP124_HUMAN	IP^44^, ProP-PD^15^
AGRETTV	1		KIF1B_HUMAN, isoform 3	
WKNETTV	1	1	GP123_HUMAN	IP^44^, ProP-PD^15^
EKKHTLL		18	E9PN86_HUMAN****	
VSKETPL		5	MK12_HUMAN	
SARSTDV		4	ANO9_HUMAN	Y2H^46^, ProP-PD^15^
RAISTDV		2	F163A_HUMAN	ProP-PD^15^
TSRETDL		1	KCNA5_HUMAN	Co-IP^47^, ProP-PD^15^
YRRESAI		1	KCNJ4_HUMAN	IP^48^
PGKETQL		1	SO1C1_HUMAN	ProP-PD^15^
YKKETPL		1	ANR50_HUMAN	
KGTETTL		1	S4A4_HUMAN isoform 5	
AGKTTIL		1	F8VP99_HUMAN	

Underlined are the nine peptides chosen to represent the sequence space for C-termini of ligands within the SAP97 PDZ2 interactome.

^*^The prevalence equals the number of a particular peptide among the sequenced survivors for the respective ProP-PD.

^**^The number in parenthesis equals the number of sequenced binding clones.

^***^Reference to publication, together with the method used in it, which supports that this C-terminus is a potential target of SAP97.

^****^Partial transcript of EIF3M_HUMAN^15^.
