# Supplementary material for: Improved affinity at the cost of decreased specificity: a recurring theme in PDZ-peptide interactions
Source: Sci Rep. 2016 Oct 3;6:34269. doi: 10.1038/srep34269 (PMC5046105; doi:10.1038/srep34269)
Supplement: Supplementary Information [file srep34269-s1.pdf]

SUPPORTING INFORMATION

Improved affinity at the cost of decreased specificity: a recurring theme in PDZ-peptide interactions

O. Andreas Karlsson[‡], Gustav N. Sundell[§], Eva Andersson[‡] Ylva Ivarsson[§*], and Per Jemth[‡*]

[‡]Department of Medical Biochemistry and Microbiology, Uppsala University, BMC Box 582, SE-75123 Uppsala, Sweden.

§Department of Chemistry-BMC, Uppsala University, Box 576, SE-751 23 Uppsala, Sweden.

[*] Corresponding author: Ylva.Ivarsson@kemi.uu.se and Per.Jemth@imbim.uu.se

CONTENTS

Supplementary Tables S1-S3
Supplementary Figures S1-S3.

Supplementary Table S1. Tissue expression and subcellular localization for the protein corresponding to the respective C-terminal peptide. For comparison, SAP97 is included at the bottom row.

Peptide	Uniprot*	Human protein atlas**
KRKETLV	ARHG8_HUMAN, *Neuroepithelial cell-transforming gene 1 protein (also known as Net1).*	Cytoplasmic positivity in all tissue groups except endocrine, bone marrow & immune system, pancreas and skin.
RSISTDV	F163B_HUMAN, *Protein FAM163B.*	RNA: cerebral cortex (enriched) and adrenal gland.
NSKETVV	MARH3_HUMAN, *E3 ubiquitin-protein ligase MARCH3.*	Cytoplasmic and membranous positivity in all but female tissues.
VSKETPL	MK12_HUMAN, *Mitogen-activated protein kinase 12 (also known as p38γ).*	Cytoplasmic and nuclear positivity in all but skin tissue.
SARSTDV	ANO9_HUMAN, *Anoctamin-9.*	Cytoplasmic and/or nuclear positivity in all but pancreas or adipose & soft tissue.
TSRETDL	*KCNA5_HUMAN, Potassium voltage-gated channel subfamily A member 5 (also known as Kv1.5).*	Membranous positivity in most glandular cells. Not in adipose & soft tissue.
YRRESAI	KCNJ4_HUMAN, *Inward rectifier potassium channel 4 (also known as Kir2.3).*	RNA: cerebral cortex and heart muscle.
PGKETQL	SO1C1_HUMAN	RNA: enriched in cerebral cortex
	DLG1_HUMAN, *SAP97 (also known as hDLG1).*	Ubiquitous membranous and cytoplasmic positivity.

*Entry code followed by protein name in italic letters.
**Positivity with antibody staining among the 13 tissue groups as well as the subcellular localization of it. The evidence at transcript level is listed when there is no such for the protein. Source: www.proteinatlas.org.

Supplementary Table S2. The two measured experimental parameters, the rate constants of association (k_{on}) and dissociation (k_{off}), as well as the calculated K_d value and the change in specificity for PDZØ9 towards the RRRETQV peptide, for the binding between PDZ variant and peptide.

	k_{on} (µM^{-1}s^{-1s})*				k_{off} (s^{-1})*				K_d (µM)*				
Peptide	pWT PDZ2	PDZØ9	pWT PDZ2 L391F	pWT PDZ2 K392M	pWT PDZ2	PDZØ9	pWT PDZ2 L391F	pWT PDZ2 K392M	pWT PDZ2	PDZØ9	pWT PDZ2 L391F	pWT PDZ2 K392M	Change in specificity for each peptide towards RRRETQV**
KRKETLV	6.4 ± 0.06	8.0 ± 0.06	7.4 ± 0.06	7.2 ± 0.02	3.0 ± 0.03	0.6 ± 0.005	0.6 ± 0.005	3.4 ± 0.01	0.47 ± 0.006	0.08± 0.001	0.08 ± 0.001	0.47 ± 0.002	1.1 ± 0.04
RSISTDV	21 ± 0.5	17 ± 0.2	24 ± 0.3	17 ± 0.3	26 ± 0.3	4.3 ± 0.06	4.3 ± 0.05	31 ± 0.3	1.2 ± 0.03	0.25 ± 0.005	0.18 ± 0.003	1.8 ± 0.04	1.3 ± 0.06
NSKETVV	13 ± 0.1	10 ± 0.07	13 ± 0.09	9.7 ± 0.07	8.6 ± 0.07	1.6 ± 0.01	1.9 ± 0.01	7.7 ± 0.18	0.66 ± 0.007	0.16 ± 0.002	0.15 ± 0.001	0.79 ± 0.01	1.6 ± 0.05
EKKHTLL	12 ± 0.5	14 ± 0.1	11 ± 0.08	11 ± 1.7***	112 ± 1.2	7.9 ± 0.04	7.1 ± 0.05	157 ± 2.0	9.3 ± 0.4	0.56 ± 0.005	0.65 ± 0.007	14 ± 2	0.40 ± 0.02
SARSTDV	16 ± 0.2	13 ± 0.1	18 ± 0.1	12 ± 0.1	26 ± 0.3	3.4 ± 0.06	4.0 ± 0.05	25 ± 0.3	1.6 ± 0.03	0.26 ± 0.005	0.22 ± 0.003	2.1 ± 0.03	1.1 ± 0.04
VSKETPL	40 ± 2.2	8.4 ± 0.06	11 ± 0.1	24 ± 2.2	154 ± 3.0	6.3 ± 0.2	8.8 ± 0.2	150 ± 3.0	3.9 ± 0.2	0.75 ± 0.02	0.80 ± 0.02	6.3 ± 0.6	1.3 ± 0.09
TSRETDL	33 ± 1.1	15 ± 0.2	18 ± 0.4	15 ± 0.5	71 ± 0.5	2.6 ± 0.04	3.8 ± 0.06	62 ± 0.9	2.2 ± 0.07	0.17 ± 0.004	0.21 ± 0.006	4.1 ± 0.2	0.53 ± 0.03
YRRESAI	19 ± 0.5	22 ± 0.2	19 ± 0.1	25 ± 0.7	87 ± 0.5	17 ± 0.2	15 ± 0.2	105 ± 0.5	4.6 ± 0.1	0.77 ± 0.01	0.79 ± 0.01	4.2 ± 0.1	1.1 ± 0.05
PGKETQL	18 ± 0.6	16 ± 0.2	20 ± 0.3	18 ± 0.6	51 ± 1.8	3.9 ± 0.03	5.0 ± 0.02	48 ± 2.3	2.8 ± 0.1	0.24 ± 0.004	0.25 ± 0.004	2.7 ± 0.2	0.56 ± 0.03
RRRETQV	8.0 ± 0.04	9.8 ± 0.05	8.7 ± 0.04	9.0 ± 0.04	3.2 ± 0.04	0.6 ± 0.015	0.6 ± 0.01	3.3 ± 0.02	0.40 ± 0.005	0.06 ± 0.002	0.07± 0.001	0.37 ± 0.003	1

*Value±standard error (k_{on} and k_{off}); the error for K_d is the propagated standard error ($K_d = k_{off}/k_{on}$)

**Calculated as: $(K_d^{\text{PDZØ9:peptide}}/K_d^{\text{PDZØ9:RRRETQV}})/(K_d^{\text{pWT PDZ2:peptide}}/K_d^{\text{pWT PDZ2:RRRETQV}})$

***Calculated as the mean of two experiments. Standard error = standard deviation/$2^{0.5}$.

Supplementary Table S3. Calculated coupling free energies, $\Delta\Delta\Delta G_c$, for the interaction between the two positions 391 and 392, at the transition and bound state, respectively, for the PDZØ9-peptide interaction.

	$\Delta\Delta\Delta G_c$ (kcal mol^{-1})	
Peptide	At transition state	At bound state
KRKETLV	-0.023 ± 0.008	0.048 ± 0.012
RSISTDV	-0.076 ± 0.02	0.024 ± 0.023
NSKETVV	0.017± 0.008	0.052 ± 0.013
EKKHTLL	0.19 ± 0.09	0.32 ± 0.09
SARSTDV	-0.021± 0.01	0.048 ± 0.02
VSKETPL	0.14 ± 0.061	0.31 ± 0.07
TSRETDL	0.34 ± 0.03	0.48 ± 0.03
YRRESAI	-0.072 ± 0.023	-0.04 ± 0.025
PGKETQL	-0.13 ± 0.029	-0.02 ± 0.04
RRRETQV	0.001 ± 0.005	0.018 ± 0.02

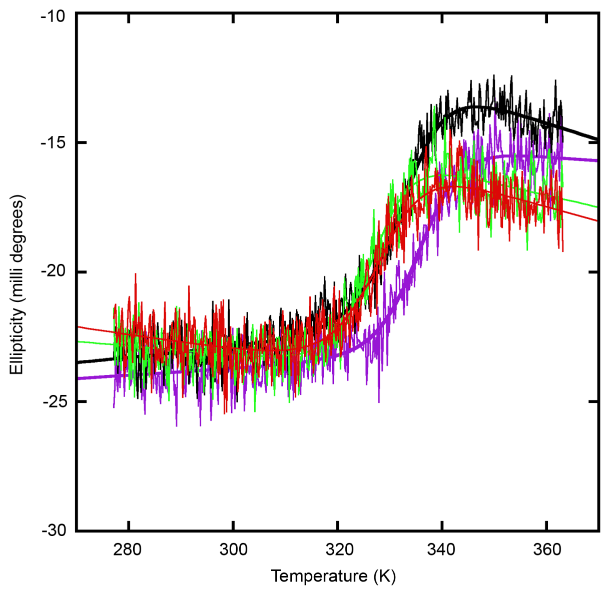

Supplementary Figure S1. Circular dichroism data for the reversible thermal denaturation of PDZ variants used in this study. The change in ellipticity for pWT PDZ2 (black), pWT PDZ2 L391F (green), pWT PDZ2 K392M (purple) and PDZØ9 (red) were all recorded using 30 µM protein. For an increased visualisation of the overlapping datasets, a fit to the Gibbs-Helmholtz equation for a reversible two-state

thermal denaturation is displayed with identical colour coding as the respective PDZ variant. As seen by the baseline of the folded state, they are all still well folded at 310 K (37°C).

Figure S2 part one

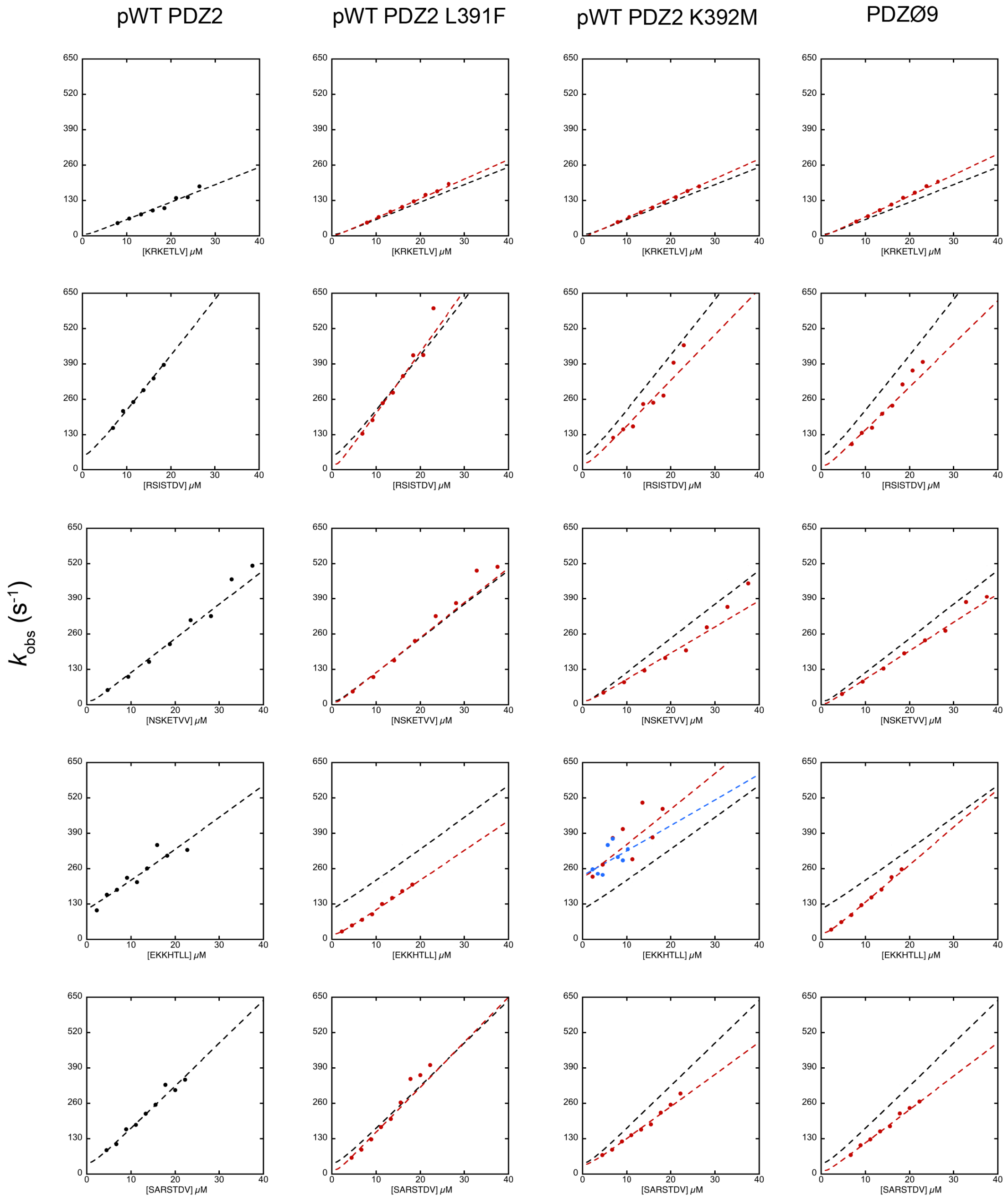

Figure S2 part two

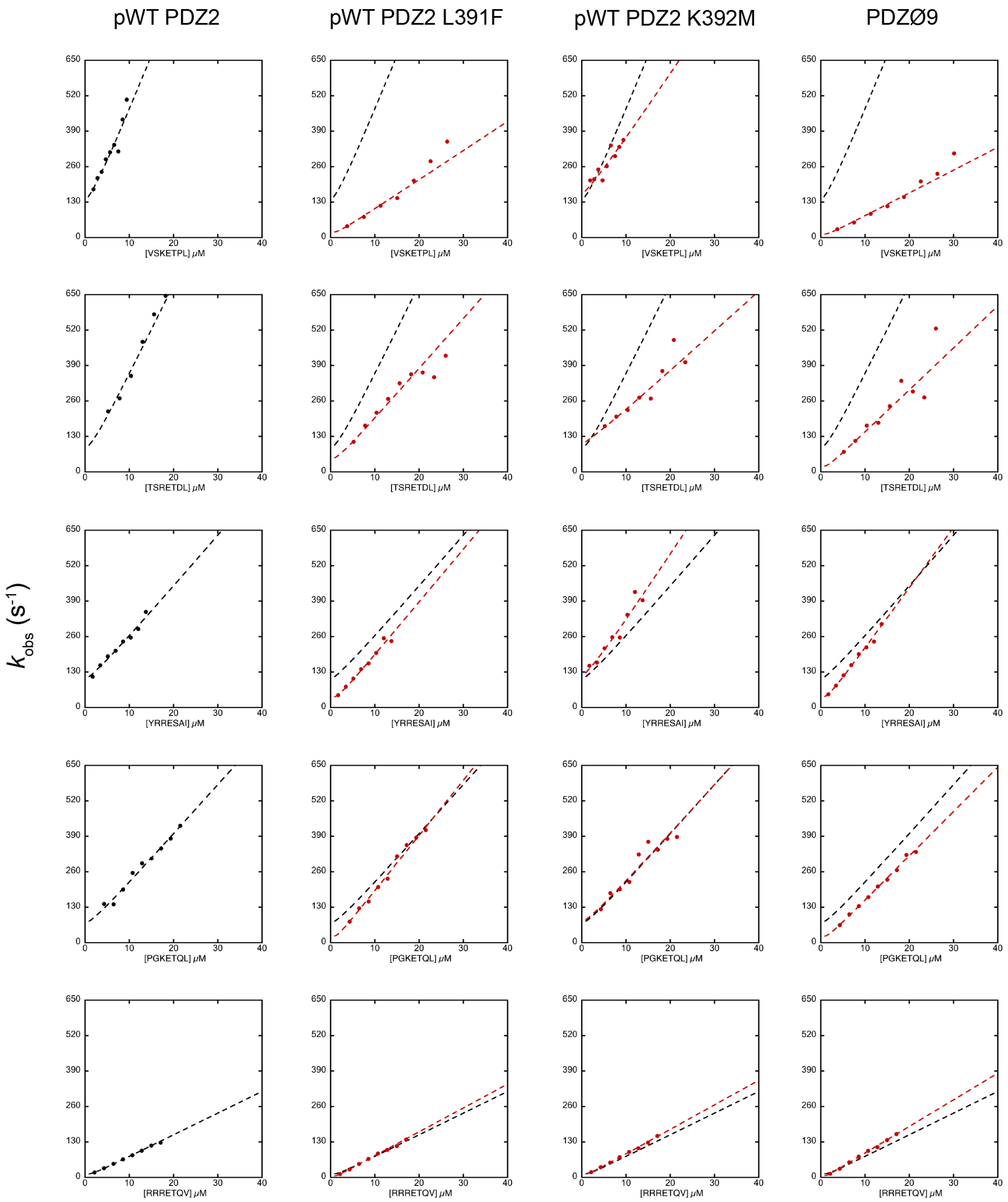

Supplementary Figure S2. Stopped-flow spectroscopy data for the binding between PDZ variant and peptide.

Data were recorded by mixing 1 μM PDZ variant with different concentrations of peptide, at conditions of 50 mM potassium phosphate, pH 7.5 and 10 °C. Each data set was fitted to the general equation for a reversible bimolecular interaction (see material and methods), and the rate constant of association, k_{on}, is equal to the slope

of the fitted dashed line at high peptide concentration. For comparison, the fit for pWT PDZ2 is displayed in black for each peptide.

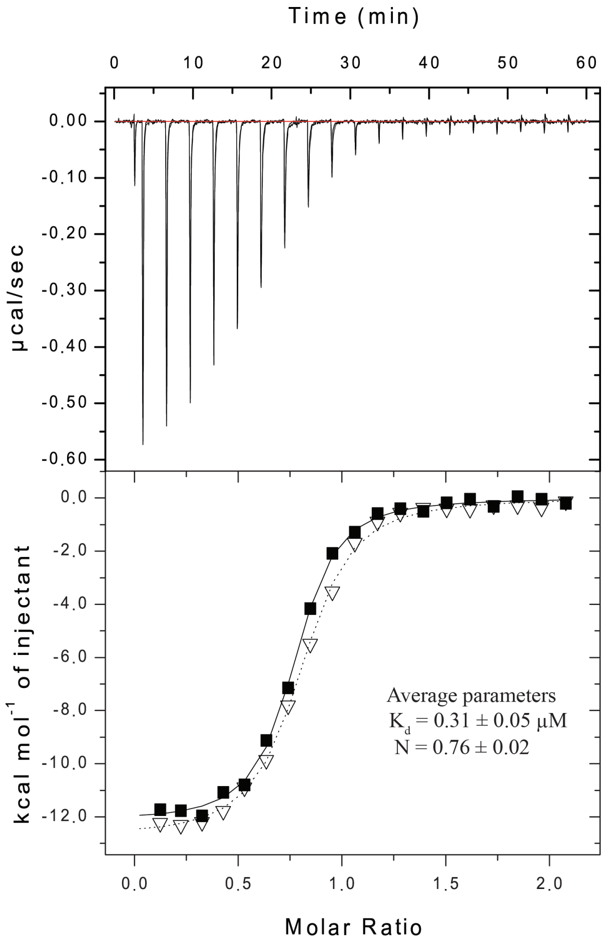

Supplementary Figure S3. ITC experiments for binding between pWT PDZ2 L391F and the PGKETQL peptide. Duplicate titrations were conducted and the resulting data sets are overlaid in the figure. For comparison, the conditions were identical to those used to collect binding data with stopped-flow spectroscopy. Average parameters from fitting are given as value ± propagated standard error from fitting.
